# IRF8-Dependent Type I Conventional Dendritic Cells (cDC1s) Control Post-Ischemic Inflammation and Mildly Protect Against Post-Ischemic Acute Kidney Injury and Disease

**DOI:** 10.3389/fimmu.2021.685559

**Published:** 2021-06-21

**Authors:** Na Li, Stefanie Steiger, Lingyan Fei, Chenyu Li, Chongxu Shi, Natallia Salei, Barbara U. Schraml, Zhihua Zheng, Hans-Joachim Anders, Julia Lichtnekert

**Affiliations:** ^1^ Department of Nephrology, Center of Kidney and Urology, The Seventh Affiliated Hospital, Sun Yat-sen University, Shen Zhen, China; ^2^ Division of Nephrology, Department of Medicine IV, University Hospital, Ludwig Maximilian University of Munich, Munich, Germany; ^3^ Walter-Brendel-Centre of Experimental Medicine, University Hospital, LMU Munich, Munich, Germany; ^4^ Institute for Cardiovascular Physiology and Pathophysiology, Biomedical Center, Faculty of Medicine, LMU Munich, Munich, Germany

**Keywords:** interferon regulatory factor 8, type I conventional dendritic cells, dendritic cells, ischemia reperfusion, acute kidney injury

## Abstract

Post-ischemic acute kidney injury and disease (AKI/AKD) involve acute tubular necrosis and irreversible nephron loss. Mononuclear phagocytes including conventional dendritic cells (cDCs) are present during different phases of injury and repair, but the functional contribution of this subset remains controversial. Transcription factor interferon regulatory factor 8 (IRF8) is required for the development of type I conventional dendritic cells (cDC1s) lineage and helps to define distinct cDC1 subsets. We identified one distinct subset among mononuclear phagocyte subsets according to the expression patterns of CD11b and CD11c in healthy kidney and lymphoid organs, of which IRF8 was significantly expressed in the CD11b^low^CD11c^high^ subset that mainly comprised cDC1s. Next, we applied a *Irf8*-deficient mouse line (*Irf8* ^fl/fl^ *Clec9a* ^cre^ mice) to specifically target *Clec9a*-expressing cDC1s *in vivo*. During post-ischemic AKI/AKD, these mice lacked cDC1s in the kidney without affecting cDC2s. The absence of cDC1s mildly aggravated the loss of living primary tubule and decline of kidney function, which was associated with decreased anti-inflammatory Tregs-related immune responses, but increased T helper type 1 (T_H1_)-related and pro-inflammatory cytokines, infiltrating neutrophils and acute tubular cell death, while we also observed a reduced number of cytotoxic CD8^+^ T cells in the kidney when cDC1s were absent. Together, our data show that IRF8 is indispensable for kidney cDC1s. Kidney cDC1s mildly protect against post-ischemic AKI/AKD, probably *via* suppressing tissue inflammation and damage, which implies an immunoregulatory role for cDC1s.

## Introduction

Ischemia-reperfusion injury (IRI) is a frequent clinical complication following kidney transplantation, volume depletion, heart failure, or major trauma, presenting as acute kidney injury (AKI). Severe IRI induced tubular necrosis implies a longer period of kidney dysfunction referred to as acute kidney disease (AKD) and, if persistent, can lead to chronic kidney disease (CKD) (1). IRI involves acute tubular epithelial cell (TEC) necrosis accompanied by a crescendo and decrescendo of sterile inflammation, referred to as “necroinflammation” (2). Necroinflammation in AKI is associated with the release of pathogen- or danger-associated molecular patterns (PAMPs or DAMPs) and the activation of kidney mononuclear phagocytes, including dendritic cells (DCs) (3, 4). Of note, the specific roles of kidney DCs and subsets in AKI/AKD remain controversial (3, 5–8).

DCs play a sentinel role between innate and adaptive immune responses. Upon kidney injury, some DCs primarily produce tumor necrosis factor-α (TNF-α) to recruit other inflammatory cells (9, 10). The antigen cross-presentation capacity of DCs can also be enhanced by PAMPs and DAMPs (10), which consequently drives naïve T cell differentiation towards cytotoxic CD8^+^ T cells or naïve CD4^+^ T cells (11, 12). Activated CD4^+^ T cells differentiate towards pro-inflammatory T_H1_ and T_H17_ cells, which stimulate macrophages, neutrophils, and other immune effectors (11–14). This avenue supports a pro-inflammatory function of kidney DCs. On the other hand, more functional studies reported protective effects of kidney DCs (15–20). This could be linked to the anti-inflammatory functions of certain DC subsets by priming naïve CD4^+^ T cells towards regulatory T cells (Tregs) to suppress inflammation (16–18, 20, 21) or enhancing cytokine pathways to improve kidney repair, including interleukin (IL)-10, IL-22, and single Ig IL-1-related receptor (3, 5, 22).

One explanation for the discrepancies on the functional role of kidney DCs may be associated with the plasticity of DCs, depending on various diseases (3, 23, 24). Kidney DCs are mixed subsets and consist of four cell subsets, namely type I conventional dendritic cells (cDC1s), type II conventional dendritic cells (cDC2s), CD64^+^F4/80^high^, and CD64^+^CD11b^high^ DCs, that differ ontogenetically, functionally, and transcriptionally (25). cDC1s derive exclusively from a single cDC precursor, whereas other DC subsets are heterogeneous (26). CD64^+^F4/80^high^ DCs are transcriptionally similar to macrophages, while CD64^+^CD11b^high^ DCs resemble cDC2s. However, the phenotype of CD64^+^ DCs still remains unclear (25). Lymphoid cDC2s consist of two subtypes with distinct functions, including the anti-inflammatory cDC2A and the pro-inflammatory cDC2B subset (27, 28). Under virus infection, cDC2s commonly share the function and gene expression with cDC1s and macrophages, collaboratively boosting CD4^+^ and CD8^+^ T cell immunity (29). This suggests that cDC1s are more homogenous than other DC subsets. In addition, the spatially and temporarily different microenvironments in diseases are responsible for the plasticity of intrarenal M1/M2 macrophages as well as DCs (8, 24). Consistently, kidney DCs are found to either play a pro-inflammatory role in adriamycin-induced nephropathy, diabetic and hypertensive nephropathy, or to be anti-inflammatory in kidney graft rejection, crescent and immune complex glomerulonephritis (3). The temporal appearance of each phenotype in different phases of single disease also varies (24).

DCs are most abundant in the interstitial compartment of the kidney and fewer than 5% of these subsets are cDC1s (25, 30). cDC1s and few plasmacytoid DCs (pDCs) exclusively arise from the C-type lectin receptor DNGR-1-expressing common DC progenitors (CDP) (31, 32). DNGR-1 is encoded by the gene *Clec9a*. In addition, cDC1s require the transcription factor *Irf8*, basic leucine zipper transcription factor ATF-like 3 (*Batf3*) and WD repeat and FYVE Domain Containing 3 (*Wdfy3*) and are marked by XCR-1, DNGR-1, and integrin CD103 (25, 26, 32, 33). DC migration also depends on chemokines and their receptors, such as C-C chemokine receptor type 7 (CCR7) (34). Functionally, cDC1s evoke IL-10-expressing Tregs to antagonize inflammatory cDC2s in crescent nephritis, or to suppress cell apoptosis in ischemic reperfusion-induced hepatic injury (18, 20, 21, 35). Furthermore, cDC1s are known for their cross-presentation capacity of antigens on MHC class I molecules to activate CD8^+^ cytotoxic T cells (36, 37). Kidney cDC1s are small subsets and the functional role of cDC1s in AKI/AKD are not well understood. Although *Xcr1*-cre, *Batf3* KO, Langerin-DTR, and *Flt3L* KO mouse lines were generated to track cDC1s, the efficiency and specificity of cDC1s reduction among these mice still need more understanding (20, 37). We generated a mouse line with *Irf8*-deficiency in *Clec9a*-expressing progenitors and hypothesized that *Irf8*-deficiency would deplete cDC1s, which may promote a pro-inflammatory immune response and consequently drive AKI/AKD progression.

## Materials and Methods

### Animals

All animal experiments were performed according to the European protection law of animal welfare and upon approval by the local government authorities Regierung von Oberbayern (Az 55.2-1-54-2532-175-2014) based on the European directive for the Protection of Animals Used for Scientific Purposes (2010/63/EU) and reported according to the ARRIVE guidelines (38). Mice were housed in groups of five under SPF condition with free access to food and water, and a 12-h light circle. Six- to eight-week-old male mice were used for experiments. The following mouse lines were used: wildtype C57BL/6 mice, *Irf8*-deficient mice (*Irf8* ^fl/fl^ *Clec9a* ^cre^ mice) as experimental group and littermates (*Irf8* ^fl/fl^ *Clec9a* ^wt^ mice) as control group (**Table S1**).

### Ischemic Reperfusion Injury (IRI Surgery)

IRI surgery was performed as previously described (39). Briefly, groups of age-matched littermate mice (*n* ≥ 3) were anesthetized to achieve analgesia, amnesia, and hypnosis prior to unilateral left kidney pedicle clamping (25 min). Body temperature was monitored by online rectal temperature recording during the whole surgery process. Following kidney pedicle clamping and clamping removal, successful reperfusion was assessed by color change from pale (ischemia) to the original color. Afterwards, wounds were closed (Ethicon, Belgium) and 500 μl saline applied to balance fluid loss. Anesthesia was antagonized as previously described (38). Mice were sacrificed on day 1 and 7 days after IRI. Left kidneys spleen and left kidney draining lymph nodes were collected for further analysis.

### Glomerular Filtration Rate (GFR) Measurement

We measured GFR in conscious mice before IR surgery as well as on days 1 and 7 after IR surgery (*n ≥* 3 mice/group) as described (39). Briefly, mice were anesthetized with isoflurane and the shaved neck was covered with a miniaturized image device built from two light-emitting diodes, a photodiode, and a battery (MediBeacon™ Inc., Mannheim, Germany). The whole recording period lasted 1.5–2 h after a single injection of FITC-sinistrin (i.v., 150 mg/kg body weight) (MediBeacon™Inc., Mannheim, Germany). Prior to the injection of FITC-sinistrin, the skins’ background signal was recorded for 5 min. Recorded mice were conscious and unrestrained in a single cage. After removing the image device, data were analyzed using the imaging device MPD Studio software (MediBeacon™Inc., Mannheim, Germany). GFR (μl/min per 100 g body weight) was calculated from the decrease of fluorescence intensity of FITC-sinistrin over time using a three-compartment model with linear correction (injection, plasma, and interstitial compartment, t1/2 of FITC- sinistrin), body weight of the mouse, and an empirical conversion factor (40).

### Cell Isolation

Kidneys were mashed gently and digested with 2 ml fresh D-PBS solution containing collagenase V (2 mg/ml, Sigma-Aldrich) and DNase I (500 Units/ml, Roche). Suspension was kept at 37°C for 45 min followed by homogenizing three to four times. Cold FACS buffer (D-PBS, 1% BSA, 0.1% NaN_3_) was added to stop tissue digestion. Digested tissues were homogenized and gently pressed through a 70 µm cell strainer (MACS^®^ SmartStrainers). Cell pellets were washed twice with D-PBS and kept on ice. Kidney leukocytes and tubular epithelial cells were enriched using a 30–70% Percoll (Sigma-Aldrich) gradient by centrifugation (2,000 rpm, 30 min, room temperature [RT]). Leukocytes were washed once with D-PBS, resuspended in 500 μl FACS buffer, and placed on ice for further analysis. Spleen and lymph nodes (25, 41) were gently pressed through a 70 µm cell strainer by using a 1 ml syringe and washed with FACS buffer. Erythrocytes in spleen were lysed with 2 ml red blood cell (RBC) lysis buffer (MilliQ water, 0.15 M NH_4_Cl) at RT for 10 min. After lysis, 8 ml D-PBS was added to stop lysis. Cell pellet was resuspended in 1,000 μl FACS buffer and stored on ice. Tubular epithelial cells were washed once with D-PBS and resuspended in lysis buffer for further RNA isolation.

### FACS Analysis of Leukocytes

Cell suspensions from the left kidney, spleen, and left kidney draining lymph node were used for FACS analysis. Cells were blocked with anti-mouse CD16/CD32 antibody (Fcγ III/II, 1 mg/ml, BD Biosciences) for 10 min on ice. After blocking, cells were stained with the fluorescent surface anti-mouse antibodies for 20 min at 4°C in the dark (**Table S2**). For intracellular staining of transcription factors, the fixation/permeabilization kit was performed according to manufacturer instruction (Foxp3/transcription factor staining buffer set, eBioscience™) and cells stained with the intracellular fluorescent-labeled anti-mouse antibodies using the indicated concentrations for 20 min at 4°C in the dark (**Table S2**). The cytometric acquisition was performed on FACSCanto^M^ II or LSRFortessa™ (BD Biosciences). Cell analysis, dot plots, and raw data export were completed using FlowJo software.

### Histology

Kidney tissues were embedded in paraffin and 2-μm kidney sections for periodic acid-Schiff (PAS) staining as described (40, 42). Representative images of kidney sections (cortex and outer medulla) are shown to illustrate tubular injury that displayed cast formation and tubular dilation. Injured tubular index was scored by the percentage of tubules in the corticomedullary junction that displayed cell necrosis, loss of brush border, cast formation, edema, and tubular dilation as follows: 0, none; 1, ≤10%; 2, 11–25%; 3, 26–45%; 4, 46–75%; 5, >76%. For immunostaining, we used biotinylated *L. tetragonolobus* lectin stain (Vector Labs), Tamm-Horsfall protein (THP) stain (Santa Cruz Biotechnology), anti-mouse IRF8 (Abcam), rabbit anti-mouse CD3 (Abcam), rat anti-mouse Ly6B.2 (Serotec, UK), and rat anti-mouse MHCII (I-A/I-E) (eBioscience™) (**Table S2**). All results were quantified by Image J software. To count interstitial cells, 10 cortical high-power fields (HPF) (400×). All assessments were performed by two blinded observers (CL and CS).

### Quantitative Real-Time PCR (qRT-PCR)

RNA was extracted from kidney tissue or isolated tubular epithelial cells using Pure Link RNA Mini Kit (Invitrogen™, Germany) according to the manufacturer’s protocol (40, 41). cDNA was synthesized from 2 μg of total RNA using the transcript kit (Invitrogen™, Germany). Quantitative real-time PCR (qRT-PCR) from cDNA was performed using SYBR Green dye detection system on a Light Cycler 480 (Roche, Germany). All samples were normalized to 18s rRNA. The sequences of gene-specific primers (300 nM; Metabion, Martinsried, Germany) are listed (**Table S3**).

### Blood Urea Nitrogen (BUN) Measurement

Serum BUN (DiaSys, Holzheim, Germany) was measured according to manufacturer’s protocol (40).

### Statistical Analysis

All data were given means ± SD. Statistical analysis of data were performed using GraphPad Prism 8 software. Data normality was checked using Shapiro-Wilk test. Comparative statistics between two unpaired groups were performed using *t-test* for parametric data and Mann Whitney test or Wilcoxon test for non-parametric data. Comparative statistics between multiple groups were performed using One-way ANOVA with Tukey’s *post-hoc* test for parametric data or Two-way ANOVA with Dunnett test for non-parametric data under Bonferroni correction. A *P*-value less than 0.05 indicated statistical significance (shown as ^*^ *P *< 0.05, ^**^ *P* < 0.01, ^***^ *P* < 0.001, ^****^ *P* < 0.0001).

## Results

### Mononuclear Phagocyte Cell Such as CD11b^low^CD11c^high^ Subset Accumulates in Kidney During Post-Ischemic AKI/AKD

**Figure 1:**
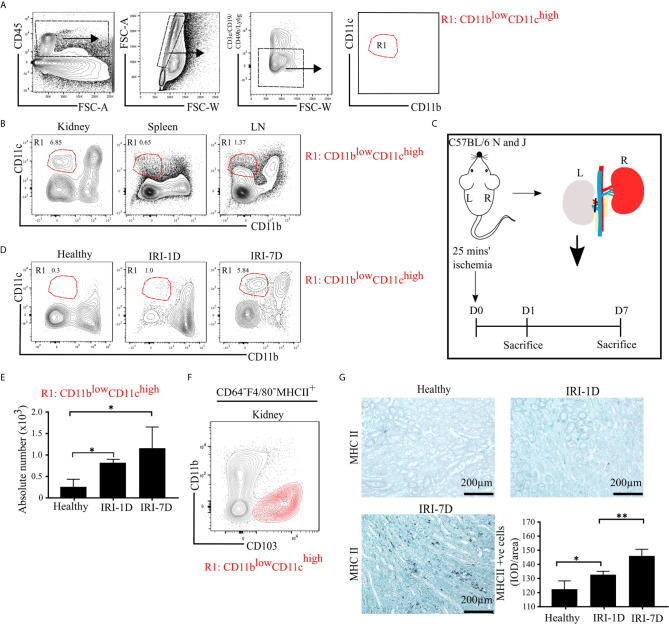
Mononuclear phagocytes, including type I conventional dendritic cells (cDC1s), accumulate in kidney during post-ischemic AKI/AKD. **(A)** Representative gating strategy of mononuclear phagocytes of adult C57BL/6 mice. Of the CD45^+^ cells and singlets FSC-W/A, mononuclear phagocytes were identified by excluding T cells/natural killer cells/neutrophils/B cells. Mononuclear phagocytes contain one distinct subset according to the expression levels of CD11b and CD11c and was named as R1 subset CD11b^low^CD11c^high^ (red). **(B)** Identification of the mononuclear phagocyte R1 subset in healthy kidney, spleen, and kidney draining lymphoid node (LN). **(C)** Schematic of experimental set-up. Unilateral IRI was induced in C57BL/6 mice by 25 min kidney pedicle clamping. Organ harvest was taken from healthy state, day 1 (IRI-1D) and day 7 (IRI-7D) after IRI. Time point D0 represents healthy state. **(D)** Dot plots displaying phenotypic change of kidney R1 subset on healthy state, IRI-1D, and IRI-7D. **(E)** Absolute cell number of R1 subset per kidney (*n* = 4–5 mice/group). **(A–E)** Representative data of three independent experiments. **(F)** Similarity between CD103^+^ cDC1s (black contour plot) and R1 subset (CD11b^low^CD11c^high^, red contour plot) in kidney (Gating strategy is shown in **Figure S1A**). **(G)** Representative immunostaining of kidney major histocompatibility complex class II (MHCII) positive cells and quantitative analysis indicated by integrated optical density (IOD)/area (*n* = 3–4 mice/group). Bars = 200 µm. Data are means ± SD. One-way ANOVA. ^*^ *P* < 0.05; ^**^ *P* < 0.01.

To characterize mononuclear phagocytes in lymphoid organs (spleen, kidney draining lymph node [LN]) and non-lymphoid organs (kidney), we used established multi-flow cytometric analysis as previously demonstrated (43). Mononuclear phagocytes of C57BL/6 mice were identified by gating on CD45^+^ leukocytes and singlets, while excluding T cells, B cells, natural killer cells (NK cells), and neutrophils (**Figure 1A**). We classified one distinct subset of mononuclear phagocytes according to the expression levels of CD11b and CD11c as followed: R1 subset (CD11b^low^CD11c^high^), which commonly exists in healthy organs, including the kidney (**Figure 1B**).

To identify the dynamic changes of the intrarenal R1 subset (CD11b^low^CD11c^high^) during AKI/AKD, wild type C57BL/6 mice underwent IRI surgery and flow cytometric analysis was performed on days 1 and 7 after IRI (**Figure 1C**). Post-ischemic AKI/AKD was associated with a significant drop in GFR on day 1 and 7 as compared to healthy mice (**Figure S1B**). The absolute number of intrarenal CD11b^low^CD11c^high^ (R1 subset) increased during the early acute injury phase (IRI-1D) and the recovery phase (IRI-7D) (**Figures 1D, E** **)**, suggesting that the R1 subset accumulates in the kidney during post-ischemic AKI/AKD.

### R1 Subset (CD11b^low^CD11c^high^) Corresponds to Type I Conventional Dendritic Cell (cDC1)-Like Cells

To confirm the phenotype characteristic of the R1 subset (CD11b^low^CD11c^high^), we further subdivided CD45^+^CD3e^−^CD19^−^CD49b^−^Ly6g^−^ mononuclear phagocytes into CD64^−^F4/80^−^ cells to exclude macrophages and MHCII^+^ cells independent of CD11c expression (**Figure S1A**). We found similar surface marker expression pattern between kidney CD103^+^ cDC1s and the R1 subset (CD11b^low^CD11c^high^) (**Figures 1F** and **S1A**), suggesting that the R1 subset (CD11b^low^CD11c^high^) shares similar phenotypic characteristics with cDC1s. Immunohistochemistry staining confirmed that the number of intrarenal MHCII^+^ cells and mRNA expression levels of *Ccr7*, *Cd40*, *Cd80*, and *Cd86* increased over time after IRI (**Figures 1G** and **S2**). Taken together, post-ischemic AKI/AKD triggered the accumulation of mononuclear phagocytes including the R1 subset (CD11b^low^CD11c^high^), which we identified as cDC1-like cells.

### cDC1s Express Transcription Factor IRF8 in Post-Ischemic AKI/AKD

**Figure 2:**
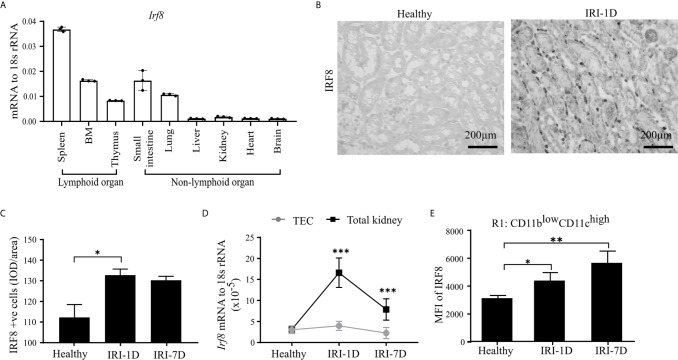
Kidney type I conventional dendritic cells (cDC1s) require transcription factor IRF8. **(A)** mRNA expression of *Irf8* in lymphoid and non-lymphoid organs from healthy C57BL/6 mice and expression level were normalized to 18s rRNA (*n* = 3 mice/group). **(B)** Representative image of IRF8 positive cells in kidney in healthy state and day 1 after IRI (IRI-1D). Bars = 200 μm. **(C)** Quantitative analysis of IRF8 positive cells by integrated optical density (IOD)/area (*n* = 5 mice/group). **(D)** Normalized mRNA expression level of *Irf8* in terms of whole kidney or isolated primary tubular epithelial cells (TECs) from mice in healthy state, IRI-1D, and IRI-7D (*n* = 5 mice/group). **(E)** Mean fluorescence intensity (MFI) of IRF8 in R1 subset (*n* = 3–5 mice/group). **(E)** Representative data of three independent experiments. Data are means ± SD. One-way ANOVA. ^*^ *P* < 0.05; ^**^ *P* < 0.01; ^***^P < 0.001.

IRF8 is terminally expressed in cDC1s and important for the development of cDC1s lineage in the bone marrow (43–45). However, the expression pattern of IRF8 in intrarenal DCs in health and AKI/AKD still remains unknown. To investigate this, we first determined the mRNA expression levels of *Irf8* in lymphoid and non-lymphoid organs of healthy wild type C57BL/6 mice, and found higher *Irf8* mRNA expression levels in spleen, bone marrow, thymus, small intestine, and lung, while the *Irf8* mRNA level in the kidney was lower and comparable to that in liver and heart (**Figure 2A**). Upon IRI, the number of IRF8^+^ cells significantly increased in the tubulointerstitium (**Figures 2B, C** **)**. This was consistent with increased *Irf8* mRNA expression levels within total kidneys after IRI, but not in isolated TECs (**Figure 2D**). Flow cytometry revealed that the MFI of IRF8 among cDC1-like R1 cells (CD11b^low^CD11c^high^) as well as monocytes significantly increased over time after IRI (**Figures 2E** and **S3A, B**). We also found that the MFI of IRF8 in cDC1-like R1 cells (CD11b^low^CD11c^high^) was significantly higher than that in monocytes. Thus, kidney cDC1-like cells express and require IRF8 during post-ischemic AKI/AKD.

### Fewer cDC1s Accumulate in Post-Ischemic *Irf8*-Deficient Mice

**Figure 3:**
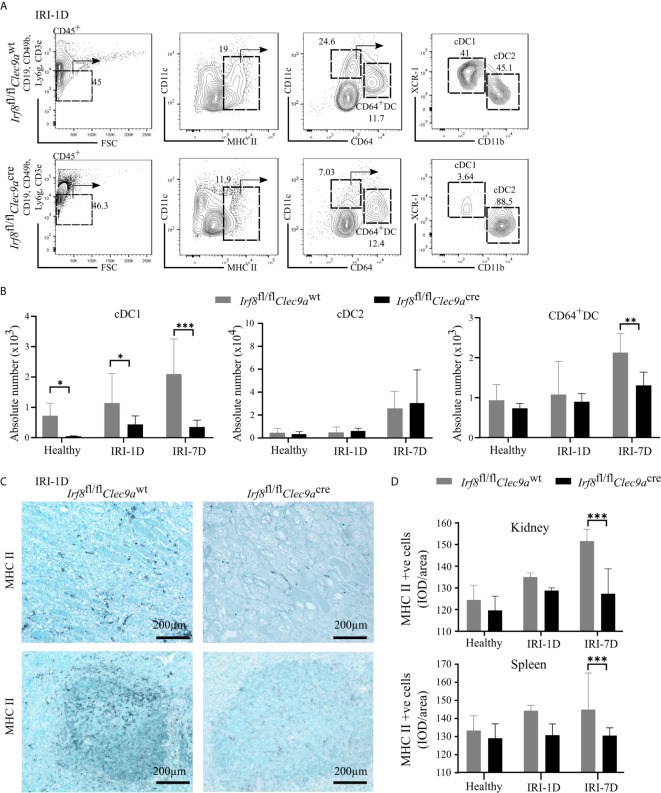
Impaired accumulation of type I conventional dendritic cells (cDC1s) in *Irf8*-deficient mice after IRI. IRI was induced in *Irf8*-deficient mice (*Irf8* ^fl/fl^ *Clec9a* ^cre^ mice) and control mice (*Irf8* ^fl/fl^ *Clec9a* ^wt^ mice). **(A)** Representative gating strategy of kidney DC subsets on day 1 after IRI (IRI-1D) (gated on live CD45^+^CD19^−^CD49b^−^CD3e^−^Ly6g^−^MHCII^+^ cells). cDCs (such as cDC1s and cDC2s) and CD64^+^ DCs were identified according to specific markers (CD11c, CD64, XCR-1, and CD11b). **(B)** Absolute cell numbers of cDC1, cDC2, and CD64^+^ DCs per kidney in healthy mice and mice after IRI. **(C)** Representative images of MHCII positive cells distributing in kidney sections (upper row) and splenic sections (bottom row) on IRI-1D. Bars = 200 μm. **(D)** Quantitative analysis of MHCII positive cells in mice in healthy state and after IRI (*n* = 3–5 mice/group). Data are means ± SD. Two-way ANOVA. ^*^ *P* < 0.05; ^**^ *P* < 0.01; ^***^P < 0.001.

To confirm whether the specific depletion of *Irf8* could reduce the accumulation of kidney cDC1s in AKI/AKD, we generated a *Irf8*-deficient model using *Irf8* ^flox/flox^ *Clec9a* ^cre^ mice (briefly called *Irf8* ^fl/fl^ *Clec9a* ^cre^) with a cre-lox recombination system. We identified the DC subsets according to various surface makers by established flow cytometric analysis (25). In detail, MHCII^+^ cells were divided into cDCs and CD64^+^ DCs according to the surface markers CD11c and CD64, while XCR-1 was used to classify cDC1s and CD11b to classify cDC2s (**Figure 3A**) (25). Under healthy condition, we observed that the absolute number of cDC1s was significantly reduced in the kidney of *Irf8*-deficient mice (*Irf8* ^fl/fl^ *Clec9a* ^cre^), but not that of cDC2s, CD64^+^ DCs and monocytes (**Figures 3B** and **S3C**). However, the absolute number of cDC1s remained significantly reduced (70–80%) even after IRI on days 1 and 7. While no difference was observed in the number of cDC2s, CD64^+^ DCs were reduced after IRI on day 7 (**Figure 3B**). Immunostaining revealed less infiltrating MHCII^+^ cells in kidney and spleen in *Irf8*-deficient mice compared to control mice after IRI, especially on day 7 (**Figures 3C, D** **)**. The data suggest that the deficiency of *Irf8* in *Clec9a*-expressing progenitors could deplete intrarenal accumulation of cDC1s after IRI. Of note, the absolute number and percentage of infiltrating monocytes in the kidney significantly increased in *Irf8*-deficient mice after IRI on day 1 due to the inflammatory response after IRI (**Figure S3D**). Together, in the *Irf8* ^fl/fl^ *Clec9a* ^cre^ mouse line, cDC1s are significantly depleted in steady state and after IRI. Additionally, we observed a partial reduction of CD64+DCs 7 days after IRI (25, 29).

### cDC1s Are Mildly Protective in Post-Ischemic AKI/AKD

**Figure 4:**
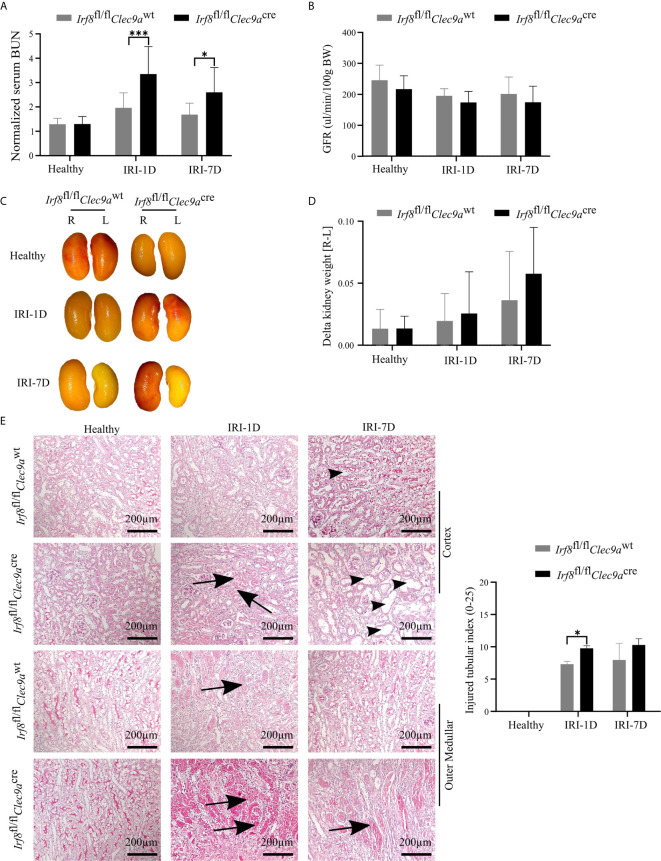
Absence of type I conventional dendritic cells (cDC1s) aggravates kidney injury during post-ischemic AKI/AKD. IRI was applied in left kidneys from *Irf8*-deficient mice (*Irf8* ^fl/fl^ *Clec9a* ^cre^ mice) and control mice (*Irf8* ^fl/fl^ *Clec9a* ^wt^ mice). **(A)** Representative values of serum blood urea nitrogen (BUN) in mice on healthy state and after IRI and normalized to respective baseline level in healthy state (*n* = 3–10 mice/group). Data were repeated twice. **(B)** GFR of *Irf8*-deficient mice and control mice from healthy state, day 1, and day 7 after IRI (*n* = 3–14 mice/group). **(C)** Representative macroscopic images of kidney atrophy. **(D)** Weight loss of left kidney [L] compared to right sham kidney [R] as illustrated as “delta kidney weight = KW_R_-KW_L_” (*n* = 3–6 mice/group). **(E)** Representative images with periodic acid-Schiff (PAS)-stained kidney sections. Tubular injury was illustrated in cortex and outer medullar and labeled with cast formation (black arrow) and tubular dilation (black arrowhead). Semiquantitative morphometry of tubular injury was shown (*n* = 3–5 mice/group). Bars = 200 μm. Data are means ± SD. Two-way ANOVA test. ^*^ *P* < 0.05; ^***^P < 0.001.

The function of cDC1s was proved controversy under crescent nephritis and adriamycin nephropathy (20, 46). To clarify the role of cDC1s in post-ischemic AKI/AKD, we applied this *Irf8*-deficient mouse model, which conditionally lacks cDC1s (**Figures 3A, B** **)**. IRI was induced in *Irf8*-deficient mice as well as control mice. We observed a significant increase of serum BUN levels, a marker of kidney excretory function (**Figure 4A**), but no significance was observed in GFR between *Irf8*-deficient mice and control mice (**Figure 4B**). Macroscopic analysis likely revealed more tubular atrophy and injury in *Irf8*-deficient mice, as indicated by a higher value of delta kidney weights (Delta kidney weight = KW_R_ – KW_L_) (**Figures 4C, D** **)**, more tubular cast formation and dilation in PAS-stained kidney sections as well as a significant higher value of injured tubular index (**Figure 4E**).

**Figure 5:**
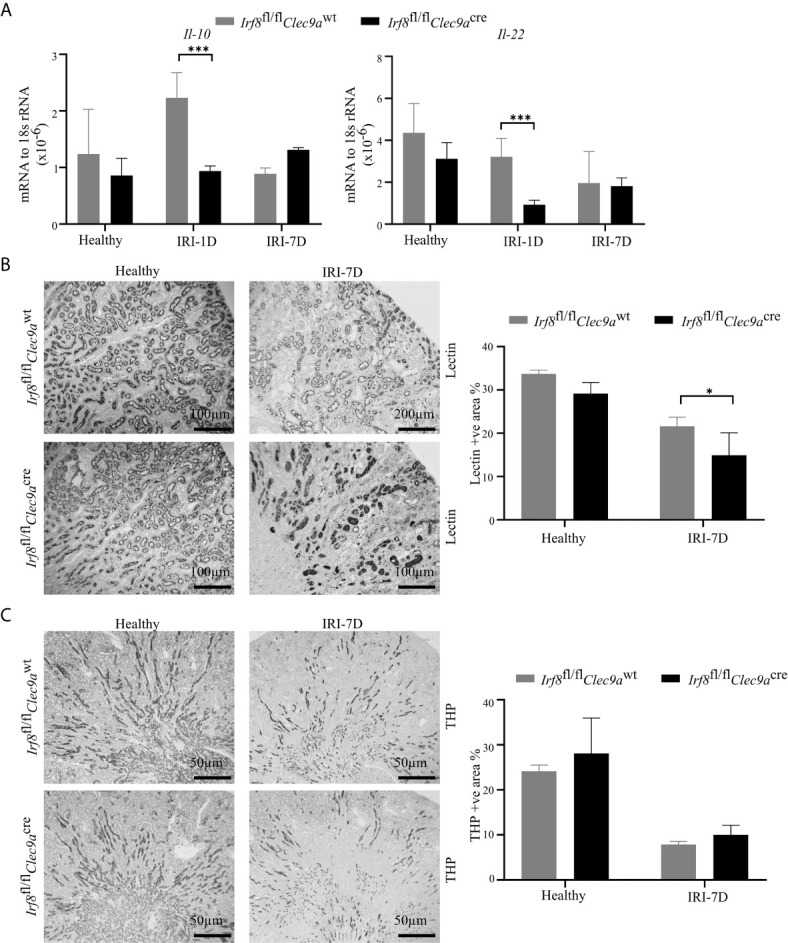
Absence of type I conventional dendritic cells (cDC1s) delays tubular recovery after IRI. IRI was induced in *Irf8*-deficient mice *(Irf8* ^fl/fl^ *Clec9a* ^cre^ mice) and control mice (*Irf8* ^fl/fl^ *Clec9a* ^wt^ mice). **(A)** Intrarenal mRNA expression of the regeneration genes interleukin-10 (*Il-10*) and *Il-22* in mice in healthy state and after IRI (*n* = 3–6 mice/group). Representative pictures of **(B)** *Lotus tetragonolobus lectin* (Lectin)-stained kidney sections for proximal tubule (Bars = 100 μm) and **(C)** Tamm-Horsfall protein (THP)-stained kidney sections for distal tubule (Bars = 50 μm) in healthy mice and 7 days after IRI. Quantitative assessment of living tubule per kidney (*n* = 3–5 mice/group). Data are means ± SD. Two-way ANOVA test. ^*^ *P* < 0.05; ^***^P < 0.001.

Intrarenal DCs also accelerate tubular regeneration and recovery by secreting IL-22 or upregulating the expression of IL-10 in AKI (5, 20). Consistent with that, we found decreased mRNA expression levels of *Il-22* and *Il-10* in *Irf8*-deficient mice after IRI as compared to control mice (**Figure 5A**). To investigate tubular recovery, immunohistochemistry staining for living proximal or distal tubules was performed during the recovery phase 7 days after IRI. We found less living proximal tubules, as indicated by reduced lectin positivity in *Irf8*-deficient mice (**Figure 5B**) and no difference in living distal tubules, as indicated by quantification of Tamm-Horsfall protein (uromodulin, THP) positive area (**Figure 5C**). Thus, our data suggest that cDC1s probably protect against tissue damage structurally but play a mild protective role on kidney function.

### Reduced Anti-Inflammatory Tregs as well as Pro-Inflammatory CD8^+^ T in Post-Ischemic *Irf8*-Deficient Mice

**Figure 6:**
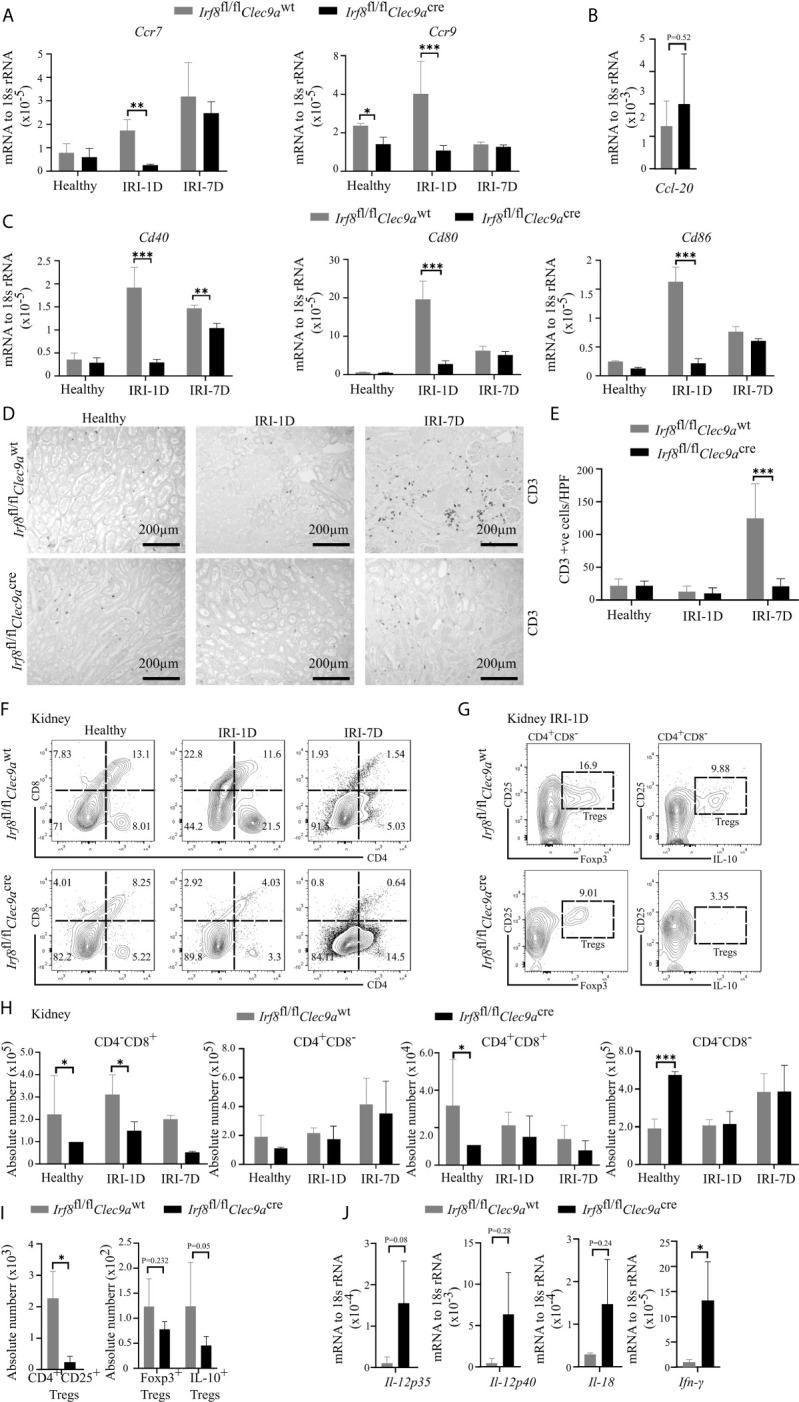
Altered T cell-related adaptive response in kidney from *Irf8*-deficient mice after IRI. IRI was induced in *Irf8*-deficient mice *(Irf8* ^fl/fl^ *Clec9a* ^cre^ mice) and control mice (*Irf8* ^fl/fl^ *Clec9a* ^wt^ mice). **(A)** mRNA expression of chemokine receptors *C-C chemokine receptor type 7* (*Ccr7*) and *Ccr9* in kidney tissues from mice in healthy state and after IRI normalized to 18s rRNA. **(B)** mRNA expression of CC-chemokine ligand-20 (*Ccl-20*) in kidney tissues on day 1 after IRI (IRI-1D) and normalized to 18s rRNA. **(C)** mRNA expression of co-stimulatory molecules *Cd40*, *Cd80*, and *Cd86* in kidney tissues from mice in healthy state and after IRI and normalized to 18s rRNA. **(A–C)** *n* = 3–5 mice/group. **(D)** Representative CD3^+^ T cell immunostaining from paraffin-embedded kidneys in mice on healthy state, IRI-1D, and IRI-7D. Bars = 200 μm. **(E)** Number of kidney CD3^+^ T cells manually counted per HPF (*n* = 3–5 mice/group). **(F)** Gating strategy of kidney T cells (gated on live CD45^+^CD19^−^CD49b^−^Ly6g^−^CD3^+^ cells) and their subsets according to CD4/CD8 expression on healthy state, IRI-1D, and IRI-7D. **(G)** Gating strategy of kidney Tregs (gated on live CD45^+^CD19^−^CD49b^−^Ly6g^−^CD3^+^CD4^+^CD8^−^CD25^+^ cells) on IRI-1D. **(H)** Absolute cell number of kidney T cell subsets (*n* = 3–8 mice/group). **(I)** Absolute cell numbers of kidney CD4^+^CD25^+^ Tregs, Foxp3^+^ Tregs, and Interlukin-10 (IL-10)^+^ Tregs on IRI-1D (*n* = 3–4 mice/group). **(J)** Gene expression of T_H1_ related cytokines on IRI-1D and normalized to 18s rRNA. See full term for each gene abbreviation in **Table S3**. *n* = 3–5 mice/group. Data are means ± SD. *t-test* or two-way ANOVA. ^*^ *P* < 0.05; ^***^P < 0.001.

Kidney DCs are known for their migratory ability to activate T cells in the kidney draining lymph node *via* chemokine receptors and co-stimulatory molecules (3). In kidneys from *Irf8*-deficient mice, while the mRNA expression level of *Ccl-20* did not change on day 1 after IRI (**Figure 6B**), we observed decreased mRNA expression levels of *Ccr7*, *Ccr9*, *Cd40*, *Cd80*, and *Cd86* as compared to control mice after IRI (**Figures 6A–C**). We also found that the number of kidney CD3^+^ T cells increased with time upon AKI in sections from control mice, while *Irf8*-deficient mice had significantly less CD3^+^ T cells after IRI on day 7 as compared to control mice (**Figures 6D, E** **)**. However, no difference of CD3^+^ T cells was observed in spleen between the two groups of mice (data not shown). This suggests an impaired T cell-related adaptive immune response in *Irf8*-deficient mice upon post-ischemic AKI/AKD.

Among T cells, aortic cDC1s preferably activate the accumulation of pro-atherogenic CD4^+^ T and CD8^+^ T cells *via* the application of *Irf8*-deficient mice (43). In adriamycin nephropathy, CD103^+^ cDC1s elicit CD8^+^ T cells aggravate tissue injury, which imply that CD8^+^ T cells were pathogenic (46). To address this in AKI/AKD, our flow cytometric analysis showed that the number of kidney CD4^−^CD8^+^ T cells was selectively reduced in *Irf8*-deficient mice overtime after IRI (**Figures 6F, H** **)**, without differences in the numbers of infiltrating kidney CD4^−^CD8^−^, CD4^+^CD8^+^, and CD4^+^CD8^−^ T cells after IRI. Similar results were obtained with splenic CD4^−^CD8^+^ T cells (**Figures S4A, C**), suggesting that cDC1s likely prime pathogenic CD8^+^ T cells upon post-ischemic AKI/AKD.

CD103^+^ cDC1s can foster the accumulation of intrarenal Tregs to protect against crescent glomerulonephritis and ischemic reperfusion-induced hepatic injury (18, 20). Since we demonstrated the efficient depletion of cDC1s and impaired expression levels of co-stimulatory molecules and chemokine receptors in *Irf8*-deficient mice on day 1 after IRI, Tregs were found to be present at the same timepoint. However, in *Irf8*-deficient mice, the numbers of CD4^+^CD25^+^ Tregs including Foxp3^+^ Tregs and IL-10^+^ Tregs were reduced in kidney and spleen (**Figures 6G, I** and **S4B, D**). Together, cDC1s are required for maintaining anti-inflammatory Tregs upon post-ischemic AKI/AKD.

### Enhanced Pro-Inflammatory T_H1_-Related Response in Post-Ischemic *Irf8*-Deficient Mice

T_H1_ cells are classically associated with IFN*-*γ secretion. Their differentiation and activity are promoted by IL-12, IL-18, and IFN-γ. IFN-γ is a cytokine, which can impair cell proliferation or activate inflammatory cell death pathways. In the kidney from *Irf8*-deficient mice, we also observed increased mRNA expression levels of T_H1_-related cytokines, including *Il-12p35*, *Il-12p40*, *Il-18*, and *Ifn-γ* after IRI on day 1 (**Figure 6J**). In summary, this indicates that cDC1s probably antagonize the pro-inflammatory T_H1_ immune response to reduce kidney injury upon post-ischemic AKI/AKD.

### Increased Neutrophils and Necroinflammation in Post-Ischemic *Irf8*-Deficient Mice

**Figure 7:**
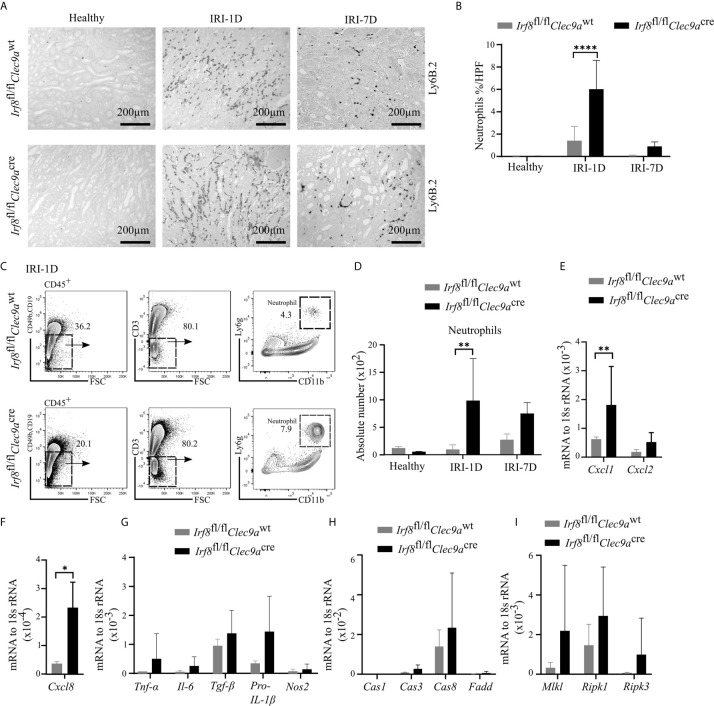
Enhanced neutrophil-related necroinflammation in *Irf8*-deficient mice after IRI. IRI was induced in *Irf8*-deficient mice (*Irf8* ^fl/fl^ *Clec9a* ^cre^ mice) and control mice (*Irf8* ^fl/fl^ *Clec9a* ^wt^ mice). **(A)** Representative images of Ly6B.2^+^ neutrophils in kidney sections from healthy mice and mice after IRI. Bars = 200 μm. **(B)** Quantification of Ly6B.2-stained neutrophils in kidney sections (*n* = 3–10 mice/group). **(C)** Gating strategy of kidney Ly6g^+^ neutrophils (gated on live CD45^+^CD19^−^CD49b^−^CD3^−^ cells) on IRI-1D by flow cytometry. **(D)** Absolute cell numbers of Ly6g^+^ neutrophils per kidney (*n* = 3–4 mice/group). **(E, F)** Normalized mRNA expression of neutrophil recruitment related, **(G)** proinflammatory, and **(H, I)** cell death-related cytokines/chemokines by qRT-PCR on IRI-1D (see full term for each gene abbreviation in **Table S3**, *n* = 3–6 mice/group). **(E–I)** Representative data repeated three times. Cas1, Caspase1; Cas3, Caspase3; Cas8, Caspase8. Data are means ± SD. *t-test* or two-way ANOVA test. ^*^ *P* < 0.05; ^**^ *P* < 0.01; ^****^ *P* < 0.0001.

Ischemic tubular necrosis involves neutrophil-related necroinflammation (2). To investigate whether the deficiency of cDC1s affects neutrophil infiltration, we performed immunohistochemistry staining of kidney sections and found a significant increased number of Ly6B.2^+^ neutrophils after IRI on day 1 in *Irf8*-deficient mice as compared to control mice (**Figures 7A, B** **)**. This was in line with the flow cytometric analysis, which showed an increased cell number of Ly6g^+^ neutrophils in *Irf8*-deficient mice after IRI on day 1 (**Figures 7C, D** **)**. In addition, we observed increased mRNA expression levels of neutrophil-related chemokines (*Cxcl1*, *Cxcl8*) and pro-inflammatory cytokines (*Tnf-α*, *Il-6*, *Tgf-β*, *pro-Il-1β*) as well as cell death-related genes (*Caspase8*, *Mlkl*, *Ripk1*, *Ripk3*) in *Irf8*-deficient mice (**Figures 7E–I**). Thus, the absence of cDC1s enhances the recruitment of neutrophils and inflammatory cell death in IRI-induced AKI/AKD.

## Discussion

We hypothesized that kidney cDC1s accumulate in post-ischemic kidneys upon AKI/AKD and that deletion of kidney cDC1s promotes a pro-inflammatory immune response and consequently drives AKI/AKD progression, all confirmed by using *Irf8*-deficient mice in our *in vivo* studies. Thus, IRF8 is an important transcription factor for differentiation and survival of kidney cDC1s, in which cDC1s and the cDC1-related immune response probably antagonize tissue injury and contribute to the recovery after post-ischemic AKI/AKD.

Previous experiments on kidney mononuclear phagocytes are confounded by the lack of specific surface markers as well as gene- and cell-specific transgenic mice to clarify the overlapping functions of cDCs with monocytes/macrophages (20, 32). So far, the surface markers CD11b and CD11c in conjunction with other markers discriminate five mononuclear phagocytes in healthy kidney (34, 47–49), of which the CD11b^low^CD11c^high^ (R1) subset matches cDC1s (34). cDC1s require the transcription factor IRF8 for their development. Thus, targeting IRF8 in DCs represents a valid strategy to clarify the role of cDC1s in homeostasis and disease. For example, deletion of *Irf8* in CD11c^+^ cells leads to a marked reduction of aortic CD11b^−^CD103^+^ DCs (cDC1s) without affecting CD11b^+^CD103^−^DCs (cDC2s) or macrophages. But CD11c is not exclusively restricted to DCs so that mouse line also affects IRF8 expression in other immune cells (29, 43, 44). In particular, monocytes require IRF8 for their transition from common monocyte progenitors (cMoP) to monocytes in the bone marrow and pDCs rely on IRF8 for cell function (44). cDCs arise from CDP, which are known to specifically express *Clec9a*. Therefore, combining *Clec9a*Cre with IRF8 floxed mice provides an improved strategy to target IRF8 in cDCs, although some pDCs are also targeted (25, 26, 32). Importantly though, *Clec9a*Cre remains restricted to DCs and is not induced on monocytes or other leukocytes in the kidney on day 3 of IRI (25). Mice lacking *Irf8* in *Clec9a*-expressing progenitors showed reduced cDC1s in steady state kidney and after IRI, indicating that IRF8 is necessary for the development of cDC1s both at steady state and upon inflammation. This is consistent with highest expression of this transcription factor in cDC1s compared to other renal DCs and macrophages (25 and **Figure 3B**). Of note, we observed a reduction of CD64^+^ DCs in *Irf8*-deficient on day 7 after IRI. It is possible that this reduction is secondary to the lack of cDC1s in this mouse model, however, since Clec9a also targets this subset, we cannot exclude a function of IRF8 in maintaining CD64^+^ DCs in IRI at this point. The use of additional mouse models, that specifically target cDC1s, such as XCR-1-diptheria toxin receptor, *Xcr1*-Cre, Langerin-Cre, or *Karma*-Cre mice may help resolve this question in future studies. Thus, we cannot exclude a contribution of *Irf8*-dependent CD64^+^ DCs on the observed effects in this context.

DCs are known for their functional contribution on T cells during AKI/AKD. For example, during kidney injury, cDCs release PAMP/DAMP-associated mediators that selectively determine the differentiation of T cells and the recruitment of these cells to the site of injury (3, 46). Our data now imply that a loss of cDC1s was associated with a lower number of T cells, including CD8^+^ T and Tregs in kidney and spleen, as well as a decrease in the expression of chemokine receptors and co-stimulatory molecules. This is in line with previous reports showing that a loss of chemokine receptors and co-stimulatory molecules ultimately impairs the migration of DCs to the kidney lymph nodes and reduces the antigen presentation capacity of DCs to T cells, e.g., CD8^+^ T and partially CD4^+^ T cells (3, 42, 50).

The protective function of cDC1s is mostly demonstrated *via* sustaining Tregs. For examples, cDC1s can drive the recruitment of IL-10-expressing CD25^+^Foxp3^+^ Tregs in nephrotoxic AKD (17), liver IRI (18), crescent glomerulonephritis (20), as well as atherosclerosis (43). The cytokine IL-22 is a member of the IL-10 family and can directly stimulate TEC regeneration following kidney injury (5, 50, 51). Consistent with previous reports, our study shows that lack of cDC1s decreased the number of Foxp3^+^ Tregs and IL-10^+^ Tregs as well as the mRNA expression of *Il-10* and *Il-22* after AKI/AKD. For their recruitment, Tregs require the chemokine receptor CCR6 and the production of CCL-20, a known CCR6 ligand (52–54). However, we did not find changes in the gene expression of *Ccl-20* in *Irf8*-deficient mice after post-ischemic AKI/AKD. Adversely, *Irf8-*deficient mice had a profound T_H1_ immune response, which promoted the conversion of naïve T into T_H1_ cells (55). Activated T_H1_ cells can also secrete CXCL8 to recruit neutrophils or IFN-γ to induce necroinflammation (55). This enhanced pro-inflammatory T_H1_ immune response was probably due to the missing immunosuppressive response of Tregs in mice (45). cDC1s excel at cross-presentation of antigens to cytotoxic CD8^+^ T cells and promote a pro-inflammatory response in lung and skin virus studies (56, 57) or adriamycin nephropathy (46). Our results showed that a reduced accumulation of kidney CD8^+^ T cells may counterbalance the interaction with protective Tregs. However, further studies are needed to address the contribution of cDC1s on Tregs and CD8^+^ T cell function.

cDCs can restrain innate immune responses (58–61). For example, DNGR-1 (encoded by gene *Clec9a*) deficiency increases neutrophilia and CXCL2 production, and consequently aggravates caerulein-induced sterile necrotizing pancreatitis during the acute phase (60). Consistently, depletion of kidney cDC1s displayed enhanced neutrophil recruitment, pro-inflammatory cytokine release, and moderate cell death, implying a role for cDC1s to regulate neutrophil-related inflammatory responses in post-ischemic AKI/AKD.

One should mention that similar to that observed in macrophage biology (61, 62), the microenvironment determines the phenotype of cDCs and the outcomes in AKI/AKD. For example, during different phases of nephrotoxic or post-ischemic AKI, MHCII^high^F4/80^high^ cells differentiate into MHCII^neg^F4/80^high^ cells, in which both may originate from DC precursor but are transcriptionally comparable to anti-inflammatory macrophages (25, 63). In post-ischemic AKI/AKD, cDC1s could contribute to an anti-inflammatory response, whereas monocyte-derived DCs differentiate into inflammatory cDC2s during infection (29). As we show herein, cDC1s comprise a small cell subset as compared to other DCs. It is possible that these cell subsets collaboratively contribute to the observed outcomes after AKI/AKD. Thus, further studies are needed to dissect the functional role of other cDC subsets and to characterize the crosstalk between cDC1s and other DC subpopulations in AKI/AKD. Meanwhile, different local microenvironment also determines the function of cDC1s. In lung and skin virus studies or adriamycin nephropathy, cDC1s dominated the MHC class I-restricted cross-presentation of both viral and self-antigens (35, 36, 46, 56, 57). In sterile kidney diseases including crescent nephritis, cDC1s most likely regulate Tregs to induce anti-inflammatory response (17, 18, 20, 21). We showed that the cDC1s-related immune response has protective effects on the outcomes after AKI/AKD. Further studies are needed to investigate the direct mechanism between cDC1s and related immune responses *in vivo*, e.g., by applying interventional studies.

In conclusion, we found that kidney cDC1s protect against post-ischemic AKI/AKD, because 1) cDC1s prime protective Tregs; 2) cDC1s enhance the expression of reparative cytokines such as IL-10 and IL-22; 3) cDC1s can antagonize the pro-inflammatory T_H1_ immune response, recruitment of neutrophils, and necroinflammation, thus leading to less kidney injury; and 4) kidney cDC1s recruit cytotoxic CD8^+^ T cells, an effect that might be counterbalanced by other anti-inflammatory cells. These findings would help to identify a functional role for intrarenal cDC1s upon AKI/AKD onset. Boosting the accumulation of cDC1s may not only potentially improve the long-term outcomes in AKI/AKD but also in other inflammatory conditions.

## Data Availability Statement

The original contributions presented in the study are included in the article/**Supplementary Material**. Further inquiries can be directed to the corresponding authors.

## Ethics Statement

The animal study was reviewed and approved by Local government authorities Regierung von Oberbayern (Az 55.2-1-54-2532-175-2014).

## Author Contributions

JL, H-JA, and ZZ designed the study and experiments. NL conducted experiments, acquired and analyzed data. SS gave suggestions for experiments design and assisted with flow cytometry. ZZ and LF provided histology samples and related data analysis. NS and BS provided *Irf8* ^fl/fl^ *Clec9a* ^cre^ mice and helped with experimental design. CL and CS performed microscopy of mouse tissue sections and related data analysis. NL, H-JA, SS, and JL wrote the manuscript. All authors contributed to the article and approved the submitted version.

## Funding

This study is supported by the Deutsche Forschungsgemeinschaft (DFG) (AN372/14-3 and AN372/24-1 to H-JA, STE2437/2-1 and STE2437/2-2 to SS, the International Program Fund for doctoral students, Sun Yat-sen University, and China Scholarship Council (CSC) 201906380147 to NL, CSC 201603250047 to CS and CSC 202008080076 to CL. Work in the Schraml lab is funded by the DFG [Emmy Noether grant: Schr 1444/1-1 and Project-ID 360372040 – SFB 1335 (project 8)] and the European Research Council (ERC-2016-STG-715182).

## Conflict of Interest

The authors declare that the research was conducted in the absence of any commercial or financial relationships that could be construed as a potential conflict of interest.
