# Supplementary material for: IRF8-Dependent Type I Conventional Dendritic Cells (cDC1s) Control Post-Ischemic Inflammation and Mildly Protect Against Post-Ischemic Acute Kidney Injury and Disease
Source: Front Immunol. 2021 Jun 21;12:685559. doi: 10.3389/fimmu.2021.685559 (PMC8255684; doi:10.3389/fimmu.2021.685559)
Supplement: Supplementary file 1 [file DataSheet_1.docx]

Supplemental Materials

[Major Resources Tables 2](#_Toc14718)

[Table S1. Animals 2](#_Toc11181)

[Table S2. Primary antibodies used in this study 3](#_Toc17757)

[Table S3. Mouse primers used in quantitative real-time PCR 5](#_Toc2696)

[Figure S1 7](#_Toc7043)

[Figure S2 8](#_Toc16782)

[Figure S3 9](#_Toc5417)

[Figure S4 10](#_Toc5910)

# Major Resources Tables

## Table S1. Animals

| **Mice line** | **Vendor or Source** | **Background Strain** | **Age and Sex** |
| --- | --- | --- | --- |
| Wild type | Charles  River Laboratories | C57BL/6N  C57BL/6J | 6-8 weeks, Male |
| *Irf8*^fl/fl^ and *Clec9a*^cre^ | Jackson Laboratories | C57BL/6 J | 6-8 weeks, Male |
| *Irf8*^fl/fl^*Clec9a*^cre^ | Walter Brendel Centre of Experimental Medicine, Munich, Germany | C57BL/6 | 6-8 weeks, Male |

**Table S1|** Animals. *Irf8*: Interferon regulatory factor 8; *Clec9a*: C-type lectin domain containing 9a.

## Table S2. Primary antibodies used in this study

| **Antibodies for flow cytometry** | | | |
| --- | --- | --- | --- |
| **Antigen** | **Fluorochrome** | **Clone** | **Manufacturer** |
| CD3 | FITC | 145-2C11 | BioLegend |
| CD3e | PerCP-Cy5.5 | 145-2C11 | BioLegend |
| CD4 | APC | GK1.5 | BioLegend |
| CD8 | PE-cy5.5 | 53-6.7 | BioLegend |
| CD11b | PE-cy7 | M1/70 | BioLegend |
| CD11c | APC-cy7 | N418 | BioLegend |
| CD19 | PerCP-Cy5.5 | 1D3/CD19 | BioLegend |
| CD25 | PE-cy7 | 3C7 | BioLegend |
| CD45 | Pacific Blue | 30-F11 | BioLegend |
| CD49b | PerCP-Cy5.5 | DX5 | BioLegend |
| CD64 | PE | X54-5/7.1 | BioLegend |
| CD103 | Brillant Violet 786 | 2E7 | BioLegend |
| F4/80 | Brillant Violet 510 | 6F12 | BD |
| Foxp3 | PE | MF23 | BD |
| IRF8 | APC | U31-644 | BD |
| IL-10 | PE | JES5-16E3 | BD |
| Ly6c | APC | HK1.4 | BioLegend |
| IgG1 kappa | APC | P3.6.2.8.1 | eBioscience^TM^ |
| Ly6g | FITC | 1A8 | BioLegend |
| Ly6g | PerCP-Cy5.5 | 1A8 | BioLegend |
| MHC II | FITC | M5/114.15.2 | BioLegend |
| XCR-1 | Brillant Violet 650 | ZET | BioLegend |
| **Antibodies for immunohistochemistry** | | | |
| **Antigen** | **Host** | **Class/Clone** | **Manufacturer** |
| CD3 | Rabbit | Polyclonal | Abcam |
| IRF8 | Rabbit | Polyclonal | Abcam |
| Ly6B.2 | Rat | Polyclonal | AbD Serotec |
| MHC II (I-A/I-E) | Rat | Monoclonal | eBioscience™ |

**Table S2|** Primary antibodies used in this study. CD: Cluster of differentiation molecule; MHC II: Major histocompatibility complex II; IRF: Interferon regulatory factor; XCR-1: X-C motif chemokine receptor-1; Foxp3: Forkhead box p3; IL-10: Interlukin-10.

## Table S3. Mouse primers used in quantitative real-time PCR

| **Mouse gene** | **Forward primer 5’->3’** | **Reverse primer 5’->3’** |
| --- | --- | --- |
| *Caspase1* | TCAGCTCCATCAGCTGAAAC | TGGAAATGTGCCATCTTCTTT |
| *Caspase3* | GGAGTCTGACTGGAAAGCCGAA | CTTCTGGCAAGCCATCTCCTCA |
| *Caspase8* | ATGGCTACGGTGAAGAACTGC | TAGTTCACGCCAGTCAGG |
| *Ccl-20* | GTGGGTTTCACAAGACAGATGGC | CCAGTTCTGCTTTGGATCAGCG |
| *Ccr7* | CCCCATCCCACTTATAACTACCTCAA | TGGCAGCCTCTCACTTTCACTTTT |
| *Ccr9* | GCCATGTTCATCTCCAACTGCAC | CCTTCGGAATCTCTCGCCAACA |
| *Cd40* | ACCAGCAAGGATTGCGAGGCAT | GGATGACAGACGGTATCAGTGG |
| *Cd80* | CCTCAAGTTTCCATGTCCAAGGC | GAGGAGAGTTGTAACGGCAAGG |
| *Cd86* | ACGTATTGGAAGGAGATTACAGCT | TCTGTCAGCGTTACTATCCCGC |
| *Cxcl1* | ﻿TCCAGAGCTTGAAGGTGTTGCC | AACCAAGGGAGCTTCAGGGTCA |
| *Cxcl2* | CATCCAGAGCTTGAGTGTGACG | GGCTTCAGGGTCAAGGCAAACT |
| *Cxcl8* | GGTGATATTCGAGACCATTTACTG | GCCAACAGTAGCCTTCACCCAT |
| *Fadd* | ﻿CACACAATGTCAAATGCCACCTG | TGCGCCGACACGATCTACTGC |
| *Ifn-γ* | CAGCAACAGCAAGGCGAAAAAGG | TTTCCGCTTCCTGAGGCTGGAT |
| *Il-6* | TACCACTTCACAAGTCGGAGGC | CTGCAAGTGCATCATCGTTGTTC |
| *Il-10* | CGGGAAGACAATAACTGCACCC | CGGTTAGCAGTATGTTGTCCAGC |
| *Il-12p35* | ACGAGAGTTGCCTGGCTACTAG | CCTCATAGATGCTACCAAGGCAC |
| *Il-12p40* | TTGAACTGGCGTTGGAAGCACG | CCACCTGTGAGTTCTTCAAAGGC |
| *Il-18* | GACAGCCTGTGTTCGAGGATATG | TGTTCTTACAGGAGAGGGTAGAC |
| *Il-22* | TGGGATTTGTGTGCAAAAGCA | TAATTTCCAGTCCTGTCTTCTG |
| *Irf8* | CAATCAGGAGGTGGATGCTTCC | GTTCAGAGCACAGCGTAACCTC |
| *Mlkl* | CTGAGGGAACTGCTGGATAGAG | CGAGGAAACTGGAGCTGCTGAT |
| *Nos2* | TGGTGGTGACAAGCACATTT | AAGGCCAAACACAGCATACC |
| *pro-Il-1β* | TGGACCTTCCAGGATGAGGACA | GTTCATCTCGGAGCCTGTAGTG |
| *Ripk1* | GACTGTGTACCCTTACCTCCGA | CACTGCGATCATTCTCGTCCTG |
| *Ripk3* | GAAGACACGGCACTCCTTGGTA | CTTGAGGCAGTAGTTCTTGGTGG |
| *Tgf-β* | TGATACGCCTGAGTGGCTGTCT | CACAAGAGCAGTGAGCGCTGAA |
| *Tnf-a* | GGTGCCTATGTCTCAGCCTCTT | GCCATAGAACTGATGAGAGGGAG |
| *18s* | GGCTTCAGGGTCAAGGCAAACT | GGCTTCAGGGTCAAGGCAAACT |

**Table S3|** Mouse primers used in quantitative real-time PCR. *Ifn-γ*: Interferon gamma; *Il:* Interlukin; *Mlkl*: Mixed lineage kinase domain like pseudokinase; *Ripk*: Receptor-interacting serine/threonine-protein kinase; *Fadd*: Fas associated via death domain. *Tnf-ɑ*: Tumor necrosis factor-ɑ; *Cxcl*: Chemokine (C-X-C motif) ligand; *Ccr*: C-C Motif chemokine receptor; *Nos2*: Nitric oxide synthase 2; *Ccl-20*: CC-chemokine ligand-20.

# Figure S1

**
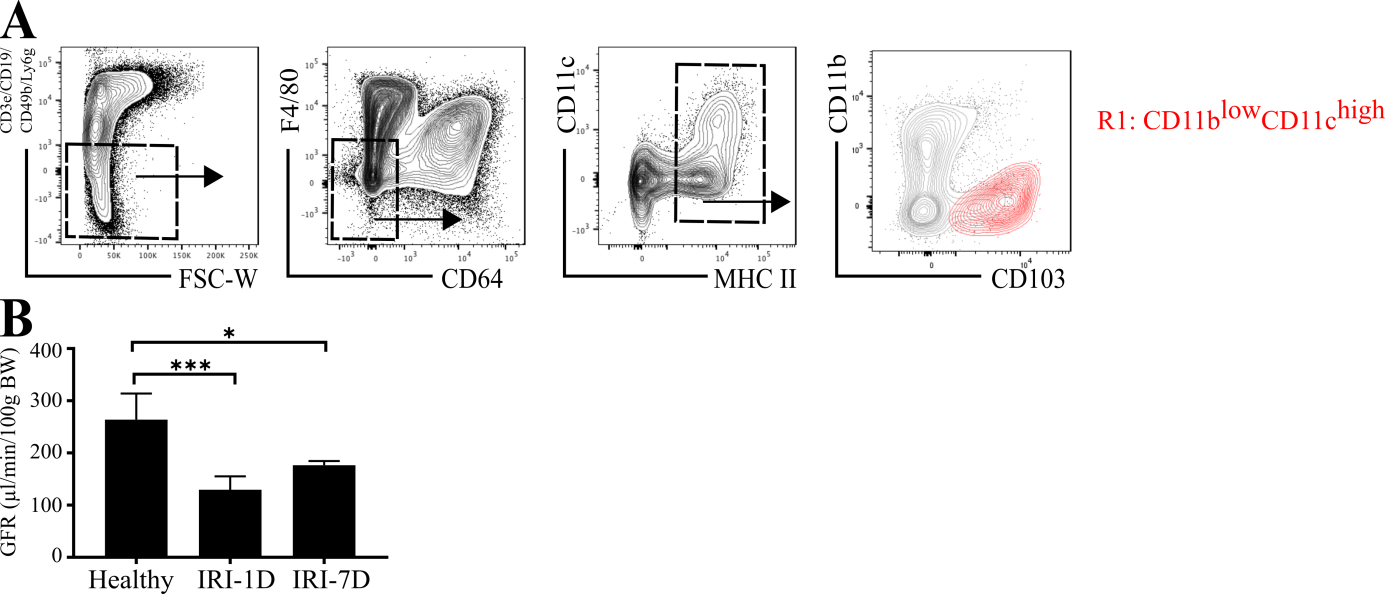
Figure S1|** Gating strategy for CD103^+^ type I conventional dendritic cells (cDC1s) in mice kidney and GFR measurement. (**A**) Representative gating strategy for comparing similarities between CD103^+^cDC1s and the R1 subset (CD11b^low^CD11c^high^) in kidney from C57BL/6 mice. In detail, mononuclear phagocytes were subdivided into CD64^-^F4/80^-^ cells to exclude macrophages. From the CD64^-^F4/80^-^ cells, MHCII^+^ cells were identified independent of the expression of CD11c, and the surface markers CD103 was used to identify kidney cDC1s. The R1 subset (CD11b^low^CD11c^high^, red contour plot) shares similarity with kidney cDC1s (black contour plot). (**B**) Glomerular filtration rate (GFR) in healthy mice and mice after IRI on day 1 and day 7 (*n* = 3-16 mice/group). Data are means ± SD. One-way ANOVA. *^*^P*<0.05; *^**^P*<0.01; *^***^P*<0.001.

# Figure S2


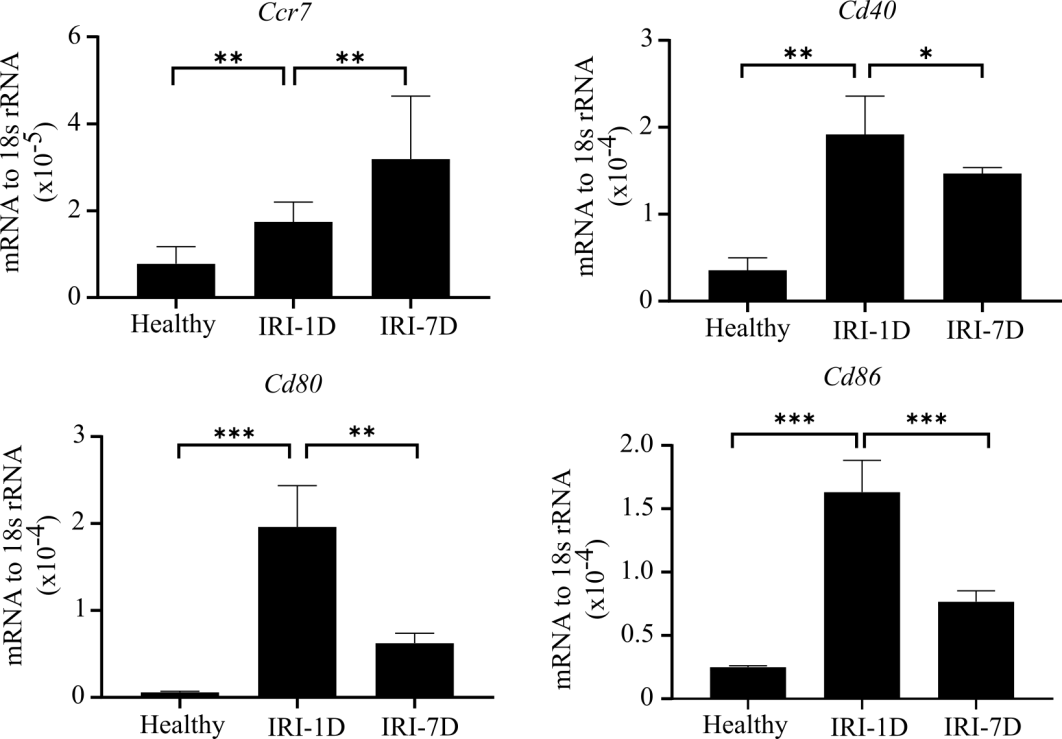


**Figure S2**| mRNA expression levels of the chemokine receptor *C-C chemokine receptor type 7 (Ccr7)* and the co-stimulatory molecules *Cd40, Cd80* and *Cd86* in kidney tissues from healthy C57BL/6 mice and C57BL/6 mice after IRI. *n* = 3-5 mice/group. Data are means ± SD. One-way ANOVA. *F*P*<0.05; ***P*<0.01; ****P*<0.001.

# Figure S3

**
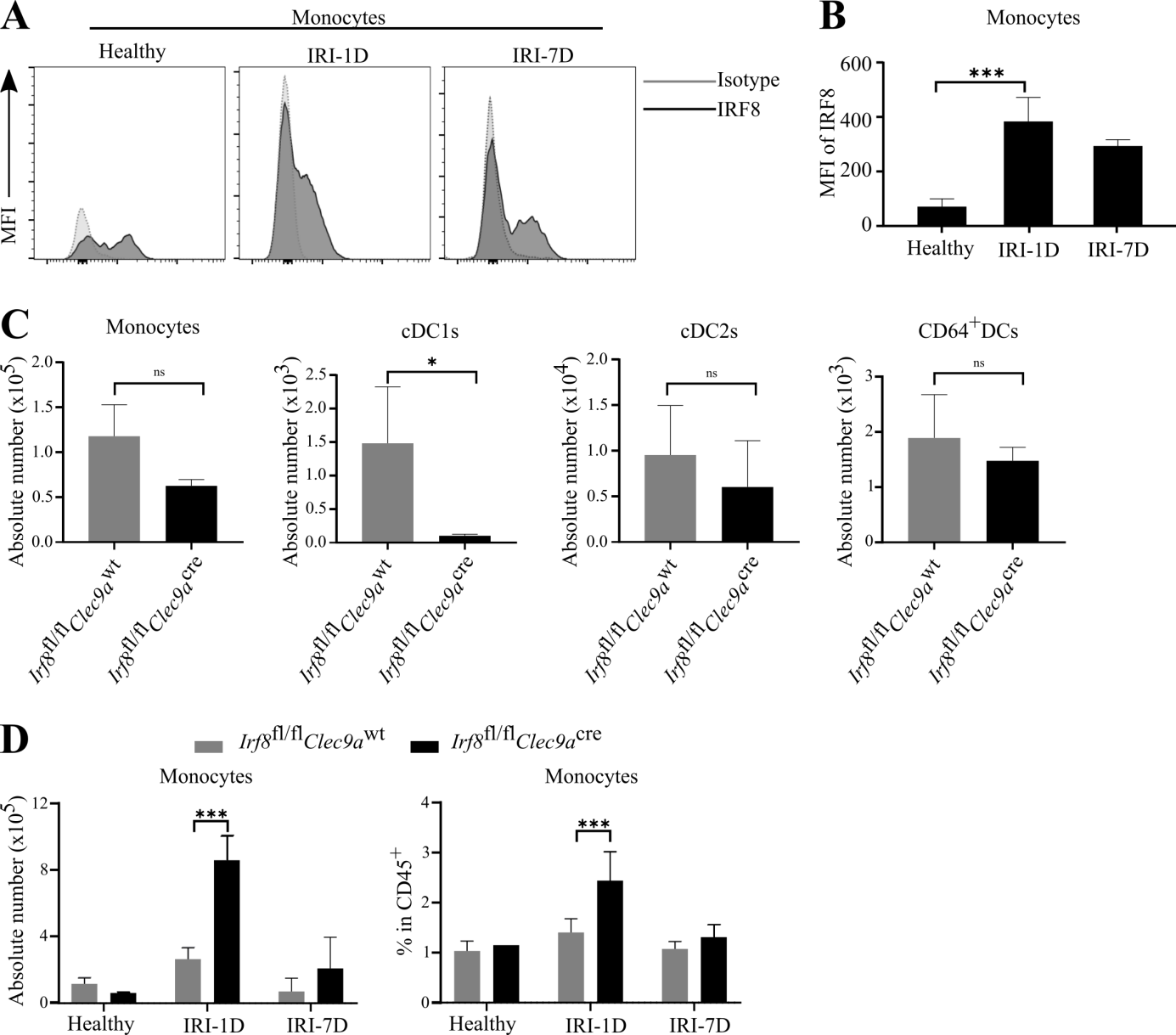
**

**Figure S3|** Effect of *Irf8*-deficiency on monocytes in kidney on healthy state and after IRI. (**A-B**) Mean fluorescence intensity (MFI) of IRF8 in intrarenal monocytes from healthy C57BL/6 mice and mice after IRI. Gray traced staining with isotype-matched control antibodies (*n* = 5-7 mice/group). Monocytes were gated on live CD45^+^CD19^-^CD49b^-^CD3e^-^Ly6g^-^CD11b^+^Ly6c^+^cells. (**C**) Absolute number of intrarenal monocytes, cDC1s, cDC2s and CD64^+^DCs in healthy *Irf8*-deficient mice (*Irf8*^fl/fl^*Clec9a*^cre^ mice) and control mice (*Irf8*^fl/fl^*Clec9a*^wt^ mice). (**D**) Absolute number and percentage of intrarenal monocytes in *Irf8*-deficient mice (*Irf8*^fl/fl^*Clec9a*^cre^ mice) and control mice (*Irf8*^fl/fl^*Clec9a*^wt^ mice) on healthy state and after IRI (*n* = 3-10 mice/group). Data are means ± SD. Two-way ANOVA. *^*^P*<0.05; *^**^P*<0.01; *^***^P*<0.001.

# Figure S4


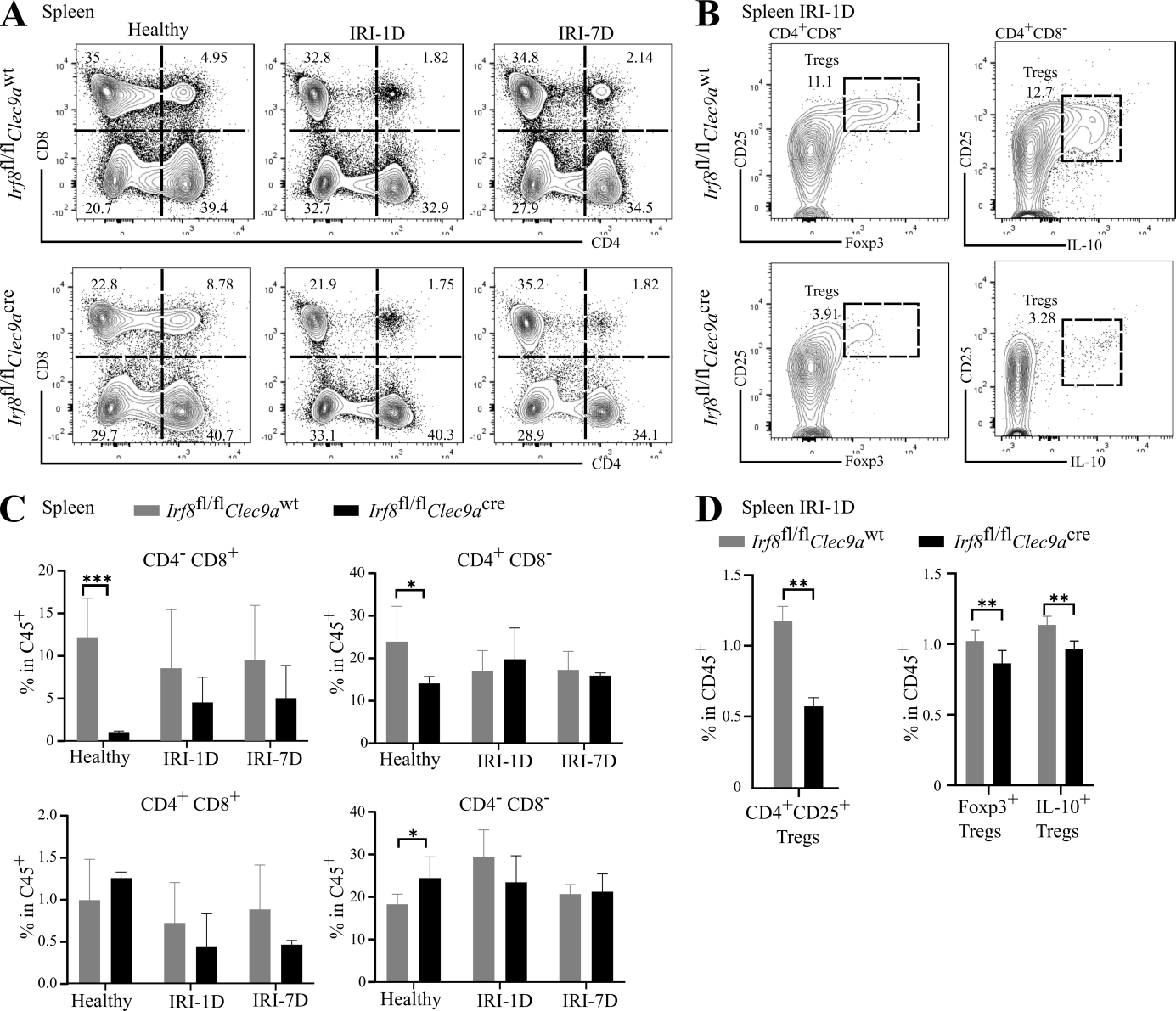


**Figure S4|** Altered T cell-related adaptive response in spleen from *Irf8*-deficient mice after IRI. IRI was induced in *Irf8*-deficient mice (*Irf8*^fl/fl^*Clec9a*^cre^ mice) and control mice (*Irf8*^fl/fl^*Clec9a*^wt^ mice). (**A**) Flow cytometry of post-ischemic spleen T cells (gated on live CD45^+^CD19^-^CD49b^-^Ly6g^-^CD3^+^ cells) and their subsets according to CD4/CD8 expression on healthy state, 1 day (IRI-1D) and 7 days (IRI-7D) after IRI. (**B**) Gating strategy of splenic regulatory T cells (Tregs) on IRI-1D (gated on live CD45^+^CD19^-^CD49b^-^Ly6g^-^CD3^+^CD4^+^CD8^-^CD25^+^ cells). (**C**) Percentage of individual T cell subsets within the splenic CD45^+^ leukocyte population on healthy state, IRI-1D and IRI-7D (*n* = 3-10 mice/group). (**D**) Percentage of splenic CD4^+^CD25^+^ Tregs including Foxp3^+^ Tregs and Interlukin-10 (IL-10)^+^ Tregs on IRI-1D (*n* = 3-5 mice/group). Data are means ± SD. Two-way ANOVA. *^*^P*<0.05; *^**^P*<0.01; *^***^P*<0.001.
